# The effects of socioeconomic status, accessibility to services and patient type on hospital use in Western Australia: a retrospective cohort study of patients with homogenous health status

**DOI:** 10.1186/1472-6963-6-74

**Published:** 2006-06-15

**Authors:** Rachael E Moorin, C D'Arcy J Holman

**Affiliations:** 1 Australian Centre for Economic Research on Health (ACERH), School of Population Health, The University of Western Australia, 35 Stirling Highway, Crawley, WA 6009, Australia; 2 Centre for Health Services Research, School of Population Health, The University of Western Australia, 35 Stirling Highway, Crawley, WA 6009, Australia

## Abstract

**Background:**

This study aimed to investigate groups of patients with a relatively homogenous health status to evaluate the degree to which use of the Australian hospital system is affected by socio-economic status, locational accessibility to services and patient payment classification.

**Method:**

Records of all deaths occurring in Western Australia from 1997 to 2000 inclusive were extracted from the WA mortality register and linked to records from the hospital morbidity data system (HMDS) via the WA Data Linkage System. Adjusted incidence rate ratios of hospitalisation in the last, second and third years prior to death were modelled separately for five underlying causes of death.

**Results:**

The independent effects of socioeconomic status on hospital utilisation differed markedly across cause of death. Locational accessibility was generally not an independent predictor of utilisation except in those dying from ischaemic heart disease and lung cancer. Private patient status did not globally affect utilisation across all causes of death, but was associated with significantly decreased utilisation three years prior to death for those who died of colorectal, lung or breast cancer, and increased utilisation in the last year of life in those who died of colorectal cancer or cerebrovascular disease.

**Conclusion:**

It appears that the Australian hospital system may not be equitable since equal need did not equate to equal utilisation. Further it would appear that horizontal equity, as measured by equal utilisation for equal need, varies by disease. This implies that a 'one-size-fits-all' approach to further improvements in equity may be over simplistic. Thus initiatives beyond Medicare should be devised and evaluated in relation to specific areas of service provision.

## Background

Australia has a universal publicly funded system of health care (Medicare), which operates in tandem with an optional 'opt up' private health sector. A principal objective of Medicare is to remove, or at least reduce, financial barriers to access to health care for all Australian residents [1]. The system is designed so that health services are accessible based on need regardless of social, locational or financial status. Such universal health insurance systems are expected to be equitable; however, a survey of community perceptions of the health systems in five countries, including Australia, found that the problems experienced by lower income groups in accessing health care persisted even in the presence of universal health insurance coverage [2].

Studies have shown that variations in service provision are often poorly explained by variations in need [3]. This phenomenon has been coined the 'inverse care law'[7], first described by Tudor Hart in 1971, whereby the availability of medical care tends to vary inversely with the need for it in the population served [7].

Numerous studies have indicated that distance to health care services is inversely associated with utilisation [4-6]. In Australia, Jong et al [8] found that people living in remote and rural areas had limited access to health services compared with those living in the metropolitan areas. Important contributing factors included geographic isolation, poor transport links, shortage of health care providers and an overall lower socioeconomic status. The authors found that in NSW people living in remote areas diagnosed with cancer were approximately 35% more likely to die as a result of their cancer over the ensuing five years compared with those living in metropolitan areas [8].

The 'inverse care law' is not followed consistently by the evidence in relation to socioeconomic disadvantage. Studies investigating the use of health services typically report that socially disadvantaged groups have higher levels of hospital admissions and medical consultations, but make less use of preventative and screening services [9]. This relationship has largely been explained by health status, whereby morbidity and premature mortality are disproportionately concentrated among the socioeconomically disadvantaged [9]. Thus, all things being equal, those with greatest need, and the socioeconomically disadvantaged, should have the greatest utilisation in an equitable health system.

Since equity (strictly speaking horizontal equity) in health care can be defined as equal treatment for equal need [10], the degree to which utilisation of the Australian hospital system is affected by socioeconomic status, locational accessibility and private admission status (the two tier system), once needs are taken into account, is a measure of its (horizontal) equity. However, the independent effects of socio-economic status, locational accessibility to services and private patient status on hospital utilisation rates have to date been difficult to evaluate due to confounding by severity of illness and comorbidity [11-14].

This study aimed to adjust for severity of illness by investigating groups of patients with relatively homogenous health status to evaluate the extent to which rates of hospital utilisation varied across socio-economic status, locational accessibility to services and private patient status when adjusted for likely confounding factors such as age, gender, race and comorbidity.

## Methods

### Sources of data

Records of all deaths occurring in Western Australia from 1997 to 2000 inclusive were extracted from the WA mortality register. For each decedent, linked records were extracted from the hospital morbidity data system (HMDS) via the WA Data Linkage System [15]. For each individual the linked data set comprised the following fields:

• From the mortality register: encrypted patient identification, age, gender, Aboriginality, cause of death, date of death and residential location recorded as postcode and collector's district (where available).

• From the HMDS: encrypted patient identification and episode number, age, gender, Aboriginality, residential location recorded as postcode and collector's district (where available), date of admission, date of separation, ICD code of principal and additional diagnoses, ICD code of principal and additional procedures, hospital type (public, private, commonwealth, other) and the payment classification recorded for each admission.

Episodes of care involving transfers between hospitals were identified for each individual in the data set using the separation and admission dates to define temporally contiguous records. These were counted as only one hospital episode. For the purposes of assigning indices of socioeconomic status, locational accessibility to services and payment classification to hospital episodes, the relevant data assigned to the first hospital record in a transfer set was the one used to assign the indices for that entire episode of care.

### Case selection and stratification

Cases were selected for the study and stratified for separate analyses according to the underlying cause of death recorded on the mortality record. The five causes of death selected for the study were colorectal cancer, lung cancer, female breast cancer, ischaemic heart disease and cerebro-vascular disease. The selection was based on national priorities and leading causes of death.

### Assignment of socioeconomic status

An index of socioeconomic disadvantage was determined for each episode of care by transformation of the collector's district (cd), or postcode where cd was unavailable, into numeric values of social disadvantage using the SEIFA (Socioeconomic Indices for Areas) index of relative disadvantage [16] based on Census data for 1996. An index of relative socioeconomic status for WA was constructed by partitioning the continuous SEIFA values into quintiles to create five ordinal categories: highly advantaged, advantaged, average, disadvantaged, and highly disadvantaged.

### Assignment of locational accessibility to services

A locational accessibility index for each episode of care was constructed using the Accessibility and Remoteness Index of Australia (ARIA) based on cd and postcodes [17]. ARIA measures accessibility to services using point location of service centres and distances by road. The classification system was that recommended by the Commonwealth Department of Health and Aged Care [18] and grouped the continuous ARIA values into five ordinal categories: major city (highly accessible), inner regional (accessible), outer regional (moderately accessible), remote and very remote.

### Assignment of private patient status

Private patient status was assigned based on the payment classification of each episode of care (public or private). Those episodes receiving a payment classification of workers compensation, motor vehicle insurance, defence force personnel and Veteran Affairs were grouped as 'other'. Individuals who had no HMDS records in a particular look-back period were classified as 'no hospital admission' and their payment status for that look-back period was assigned either based upon that of the previous HMDS record, or that of the first HMDS record, if there were no previous records. Those with no HMDS records in any look-back period were assigned to the public payment classification.

### Assignment of age at death groups

Individuals were assigned to one of five age at death groups (20 years and under, 21 to 40 years, 41 to 60 years, 61 to 80 years and 81 years and over) determined by their age at death recorded on the mortality register. These age groups were chosen because the outcomes being examined were chronic conditions whose manifestations typically occur in middle to late adulthood. Thus children and very young adults were grouped together as were the oldest old. Since the effect age *per se *was not being studied in the analysis, but age related confounding was likely, broad age groups were chosen to simplify the analysis and improve cell counts.

### Assignment of comorbid conditions

The recorded principal and additional diagnoses and procedures were used to assign co-morbid status (yes/no) to each episode of care. This was based on the observation of any of 17 co-morbid conditions [19], excluding those that came under the classification of the recorded cause of death, within 12 months of the date of admission for each particular episode of care.

### Partition of records, enumeration of total episodes of care and calculation of time at risk

For each individual the total number of episodes of care in the last year of life and the second and third years prior to death were calculated separately and recorded on the first (index) record within each look-back period. The number of days at risk for each individual within each look-back period was calculated and recorded on the index record by subtracting the total in-patient time in days (inclusive of transfer events) from the maximum available time at risk (365 days). The relevant index records, containing data on cause of death, gender, indigenous status, co-morbid binary (yes/no) indicator, age group at death, type of hospital, SEIFA category, ARIA category and admission status were partitioned from the master data set and used as input into the modelling process.

### Modelling the incidence rate ratio

After analysing the structure of the data for model appropriateness, negative binomial maximum-likelihood regression (last year of life) and the general linear model using a negative binomial variance function and a log link function (2nd and 3rd years prior to death) was used to model the incidence rate ratio of hospitalisation for each cause of death adjusted for gender, indigenous status, co-morbidity, age at death, hospital type, socio-economic status, locational disadvantage and admission status. These models were chosen over the Poisson model due to over dispersion of the data (last year of life) and over dispersion plus a high frequency of zero counts of hospital episodes (2nd and 3rd years prior to death).

## Results

### Characteristics of the data

Of the 43,812 individuals with a mortality record between 1997 and 2000, 39,099 (89.2%) had at least one hospital episode. Of the hospital episodes recorded for those individuals, 4.4% (16,752) pertained to intra hospital transfers, leaving the 39,099 individuals to utilise 364,638 separate episodes of care. Of the total number of individuals with a mortality record, 32% (13,781) were eligible for inclusion in the study because they died of one of the five conditions of interest. These patients utilised 88,499 episodes of hospital care in the last three years of life, with only 9% (1,268) not having at least one episode of hospitalisation. The distribution of individuals and episodes amongst the five causes of death for the three look-back periods are shown in table 1.

### Independent effects of socioeconomic status on the incidence rate ratio of hospitalisation

**Figure 1:**
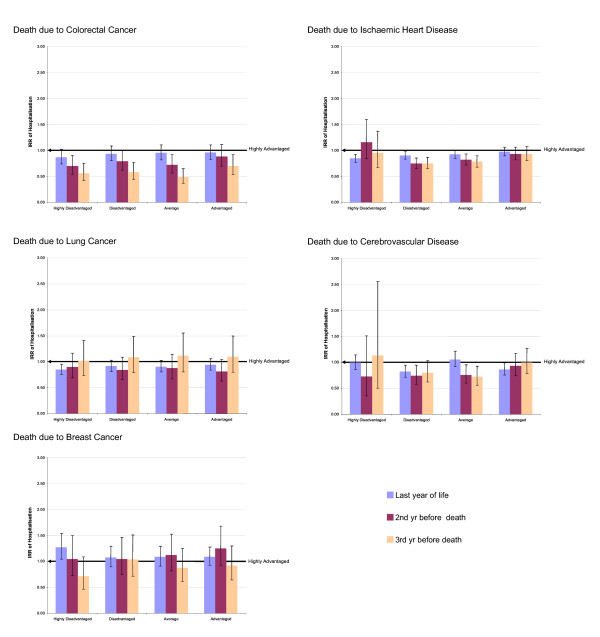
The independent effects of socio-economic disadvantage on the incidence rate of hospitalisation across five causes of death. IRR: Incidence rate ratio. All IRR's are corrected for gender, indigenous status, co-morbid conditions, hospital type, age at death, locational accessibility and admission status.

The independent effects of socio-economic status on the rate of hospitalisation across the five causes of death are shown in figure 1. The effect of socio-economic status was dependant upon both the cause and time to death. For those who died of colorectal cancer significantly higher rates of hospitalisation were generally observed in both the second and third year prior to death in highly advantaged individuals compared with the rates in individuals from lower socioeconomic groups (advantaged to highly disadvantaged). However, in the last year of life no significant difference (p ≥ 0.05) in incidence rates across levels of socio-economic disadvantage were observed.

In those who died of lung cancer, socioeconomic status was not generally associated with different hospital utilisation, except in highly disadvantaged individuals, who in the last year of life had significantly lower utilisation rates (IRR 0.85 95%CI 0.75 – 0.96). In those who died of breast cancer, highly disadvantaged individuals had a significantly increased rate of hospitalisation in the last year of life (IRR 1.27 95%CI 1.04 – 1.52). However, no significant difference (p ≥ 0.05) in rate was observed in the second or third year prior to death.

Hospital utilisation appeared to be correlated with socioeconomic status in individuals who died of ischaemic heart disease. An exception to this trend was observed in highly disadvantaged individuals, where in both the second (IRR 1.16 95% CI 0.84 – 1.60)) and third years (IRR 0.96 95% CI 0.67 – 1.37) prior to death the highest hospitalisation rates were observed. In those who died of cerebro- vascular disease, socioeconomic status showed no consistent association with utilisation.

### Independent effects of locational accessibility to services on the incidence rate ratio of hospitalisation

**Figure 2:**
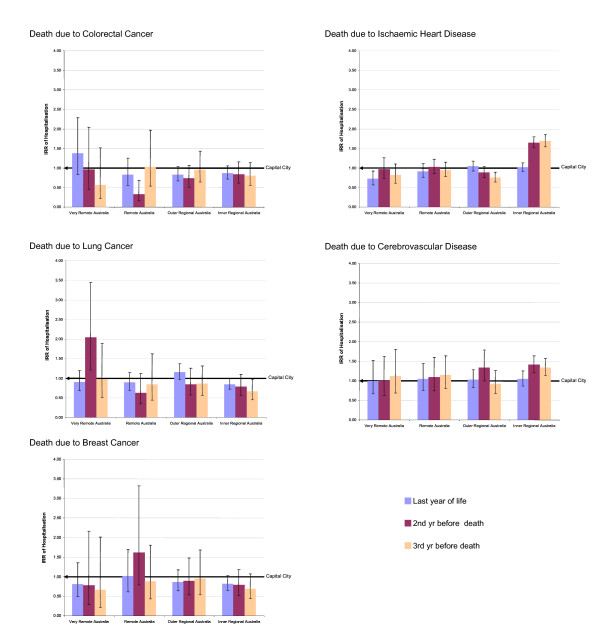
The independent effects of locational accessibility on the incidence rate of hospitalisation across five causes of death. IRR: Incidence rate ratio. All IRR's are corrected for gender, indigenous status, co-morbid conditions, hospital type, age at death, socio-economic status and admission status.

The independent effects of locational accessibility to services on hospitalisation rates across the five causes of death are shown in figure 2. Generally, locational accessibility to services had less effect on the incidence rate of hospitalisation than socio-economic status. In those who died of colorectal cancer, breast cancer or cerebro-vascular disease no significant difference (p ≥ 0.05) in hospital utilisation was observed across levels of locational accessibility. However, significantly higher rates of hospitalisation (IRR 2.05 95%CI 1.21–3.45) were observed in the second year prior to death in individuals who died of lung cancer and lived in very remote areas, and in both the second (IRR 1.65 95%CI 1.52–1.80) and third (IRR 1.69 95%CI 1.54–1.86) years prior to death in individuals who died of ischaemic heart disease and lived in inner regional areas. In addition, significantly lower hospitalisation rates (IRR 0.72 95%CI 0.56–0.92) were observed in the last year of life in individuals living in very remote areas who died of ischaemic heart disease.

### Independent effects of private patient status on the incidence rate ratio of hospitalisation

**Figure 3:**
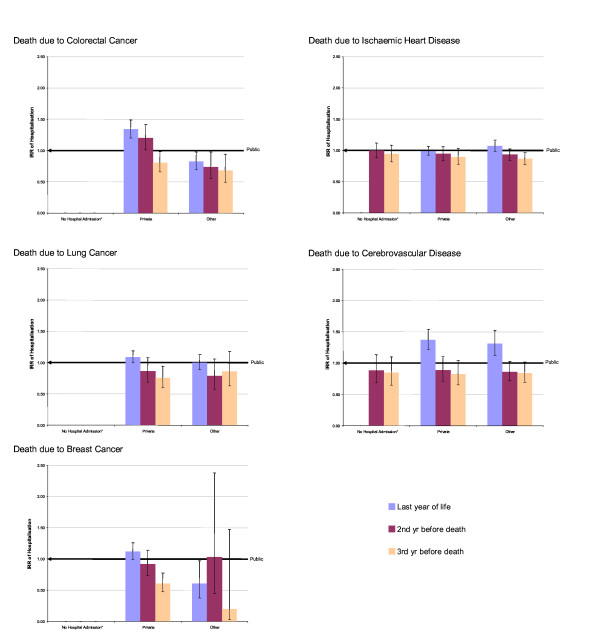
The independent effects of private patient status on the incidence rate of hospitalisation across five causes of death. IRR: Incidence rate ratio. All IRR's are corrected for gender, indigenous status, co-morbid conditions, hospital type, age at death, socio-economic status and locational accessibility. *The category no hospital admission indicates the IRR of no hospital admission for private versus public patients in each look-back period.

The independent effects of private patient status on the rate of hospitalisation across the five causes of death are shown in figure 3. Similar to the results observed for socio-economic status, the separate effect of private patient status was variable and strongly dependant upon both the cause of and time to death. The incidence rate of 'no hospital admission' in all of the three cancers for privately insured patients was significantly different (p ≥ 0.05) from the rate of no admission for public patients across all three look-back periods. However, in those who died of ischaemic heart disease and cerebro-vascular disease the rate of no admission for private versus public patients was only significantly different in the last year of life.'

For individuals who died of colorectal cancer, private admission rates were significantly higher (IRR 1.34 95%CI 1.20–1.49) in the last year of life compared with public admission rates. Whereas in the third year prior to death, private admission rates were significantly (p < 0.05) lower than public rates across all three cancers studied. For those who died of colorectal cancer admission rates for the 'other' category were significantly lower than public rates, regardless of time to death. However, this pattern was not observed for those who died of lung or breast cancer, where admission rates were generally not significantly different (p ≥ 0.05) from public rates.

In individuals who died of cerebro-vascular disease, both private and 'other' admission rates in the last year of life were significantly higher (p < 0.05) than public admission rates. While in individuals who died of breast cancer both private and 'other' admission rates were significantly lower (p < 0.05) than public rates three years and one year prior to death respectively. In contrast, individuals who died of ischaemic heart disease only showed significantly different rates of hospitalisation in the third year prior to death, where 'other' admission rates were significantly lower (p < 0.05) than public rates.

## Discussion

In this study we have examined the independent effects of socioeconomic, locational accessibility to services and private patient status on hospital utilisation rates in the last years of life in patients who died of the same underlying cause after adjustment for potential confounders.

Although the literature is replete with studies recognising that socioeconomic status has a marked effect on utilisation of health services, the majority of studies have been subject to criticism due to potential confounding by severity of illness or comorbidity. For example, in a study examining trends in patterns of hospitalisation of the New South Wales population, Walker et al [20] found that the poorest socioeconomic group had a 21% higher hospitalisation rate in 1996/1997 compared with that observed in the richest sub group, an association which was reversed for private patients. However, in that study no adjustment was made for severity of illness or comorbidity, nor was stratification by disease attempted. Thus it is difficult to determine if the differential utilisation was explained by structural differences such as variations in social support or by real differences in need, or both.

Other studies have looked at access to specialised services as a function of socioeconomic status. Coory et al [21] investigated the effects of socioeconomic status on utilisation of invasive coronary procedures in Queensland. The authors found, as has been demonstrated in numerous other studies, wide disparities in access. They concluded that free access to health care did not necessarily ensure equitable access. However, in that study there was no adjustment for severity of illness, although adjustment was made for comorbidity, for this reason the results reported by Coory et al [21] were unable to assess equity issues definitively, given that equity requires 'equal access for equal need'.

Studies that have assessed health outcome as a function of socioeconomic status have found an inverse relationship between socioeconomic status and mortality. Hall et al [22] examined the influence of social, economic and locational disadvantage on lung and breast cancer case fatality in Western Australia, finding that survival was poorer in patients treated in public hospitals, rural hospitals and relatively disadvantaged socioeconomic groups. The authors acknowledged that since no staging information was available, adjustment for severity of disease was not possible.

In this study, in addition for adjusting for demographic characteristics and comorbidity, we have at least partially adjusted for severity of illness by restricting the analyses by both the time to death and underlying cause so as to generate, as far as possible, a relatively homogenous group of patients with respect to need. Further, we have accounted for the possibility that patients from disadvantaged groups may have a shorter average duration of life-threatening illness due to more advanced disease at initial diagnosis, by undertaking separate analyses for the last, second and third year prior to death. Given that in the last year of life needs will most likely be the most homogenous, any differences between the utilisation rate in that period for a particular cause of death is more likely to be attributable to causes other than illness severity rather than differences observed in the second or third years prior to death. Therefore, comparison of utilisation trends across socio-demographic variables between look-back periods has enabled us to separate severity of illness effects from true socio-demographic effects.

Our results suggest that the independent effects of socioeconomic status differ markedly across diseases and that the consistency of effects between three and one-year observation periods before death also differs across diseases. For colorectal cancer we observed no significant socioeconomic effect in the last year of life; however, in the third year prior to death socioeconomic status had a statistically significant effect suggesting that disease severity may have confounded the effect of socioeconomic status observed earlier in the course of the disease. This would be consistent, for example, with a screening effect of higher rates of colonoscopy leading to higher and earlier hospital utilisation in more advantaged groups. For lung, breast cancer and ischaemic heart disease the reverse association was observed with respect to highly disadvantaged individuals. In the third year prior to death no statistically significant difference was observed for between highly disadvantaged and highly advantaged individuals, but in the last year of life a marked difference was observed, suggesting that socioeconomic status is a major driver of differential utilisation. Such differential hospital utilisation in terminal breast cancer patients might, for example, be due to a lower level of accessibility to hospice or home-based palliative care services.

With respect to disadvantaged, average and advantaged individuals who died of ischaemic heart disease we found that utilisation was associated with socioeconomic advantage regardless of time to death indicating that socioeconomic status was the major driver of differential utilisation. Severity of illness did not appear to confound this association because there was little difference in results across look-back periods. The effect of socioeconomic status in cerebrovascular disease was difficult to interpret since the relationship was inconsistent across disadvantage categories.

Distance to health services has long been recognised as an important factor in access to care with numerous studies describing an inverse relationship between distance and utilisation across many diseases. Jong et al [8] showed that people living in areas of Australia with limited access to services had poorer health than people living in metropolitan areas. Jones et al [5] examined the relationship between asthma mortality and access to health services, finding that asthma mortality increased with travel time to hospital. An inverse relationship between geographic proximity to services and mortality has also been demonstrated for ischaemic heart disease in Australia. Sexton et al [6] found that populations living outside capital cities had higher death rates than those living in capital cities in Australia. In addition, Hall et al [12] found that locational accessibility had a significant effect on patterns of surgical care in people with colorectal cancer and the mortality of patients with breast and lung cancer [22]. As has been the case with socioeconomic status, a major limitation of all of these studies was the lack of adjustment for severity of illness and often comorbidity as well.

Our results suggest that locational accessibility to services is generally not an independent predictor of utilisation during the years leading up to death, with the exception of in those dying from ischaemic heart disease, where we found that the incidence rate ratios were significantly different in the last year of life. These results are somewhat concordant with a study of colorectal and lung cancer which suggested that disease stage accounted for differential utilisation effects rather than locational accessibility *per se *[23].

It has been suggested that the existence of optional private health insurance in addition to the universal publicly funded Medicare system in Australia has led to a two tier system, for the rich and poor, with implications for treatment patterns and survival in economically disadvantaged groups [24]. However, proponents of the dual system argue that private health insurance is able to fund extra demands in a regulated way by providing a mechanism to ensure choice in service provision and reducing pressures on the public system [25]. Our results indicate that admission as a private patient (adjusted for hospital type, locational accessibility and socioeconomic status) did not consistently effect utilisation across all causes of death. However, we found that payment classification had a statistically significant independent effect when colorectal cancer or cerebrovascular disease was the underlying cause of death and that in both cases the effect was most marked in the last year of life when we found that private patients were more likely to be hospitalised.

### Strengths and limitations of this study

The use of probabilistically matched administrative health data made available a comprehensive patient-based data set, as opposed to a separations-based data set, enabling access to historical information on health service utilisation for the total population of WA. The study was therefore population-based rather than sample-based; allowing the health experience of all individuals who died between 01/01/1997 and 31/12/2000 to be incorporated into the modelling process. We feel this is likely to lead to greater accuracy and improved external validity compared with studies using institutional or response-based sampling from a base population [26,27].

Significant information error in our results is highly unlikely as the data used were population-based and classification regimes were applied consistently throughout the data set. Exhaustive validation research on the WA Data Linkage System [15] has shown that missing demographic data items are very uncommon (<1%). One limitation of our study is that we used time to death as a proxy for severity of illness, which was in itself a proxy for level of need. The measurement of severity of illness is a major problem when undertaking studies of this type as it cannot be readily determined from administrative data sets. Our use of time to death defined a relatively homogenous group of patients and this, combined with stratification by underlying cause of death, may have reduced the extent of confounding by stage of disease. However, the level of control of confounding was likely to have been incomplete.

## Conclusion

Our study has demonstrated that within a health system that already supports universal access through Medicare, the Australian hospital system is not equitable since equal need does not equate to equal utilisation. The largest inequity appears to be related to socioeconomic status, while a moderate level of inequity exists in relation to private patient status. Our results allude to marked variations in the influence of socioeconomic, locational and private patient status across causes of death, suggesting that equity in hospital utilisation is not universal but rather is disease specific, a finding that has not been identified previously in the literature.

Since it appears that the magnitude and direction of socially-determined differences in health care utilisation are both inconsistent and disease-specific. A 'one-size-fits-all' approach to further improvements in equity may be over simplistic. Thus initiatives beyond Medicare should be devised and evaluated in relation to specific areas of service provision.

## Competing interests

The author(s) declare that they have no competing interests.

## Authors' contributions

REM participated in the design of the study, performed the statistical analysis, interpreted the data and drafted the manuscript. CDJH Conceived of the study, participated in its design and coordination and helped to interpret the data and draft the manuscript. All authors read and approved the final manuscript.

## Pre-publication history

The pre-publication history for this paper can be accessed here:



## Figures and Tables

**Table 1:** The distribution of individuals and episodes of hospitalisation across the five cause of death categories and three look-back periods

			**Last year of life**		**2**^nd^**year prior to death**		**3**^rd^**year prior to death**	
**Cause of death**	**Individuals**		**Episodes**		**Episodes**		**Episodes**	
	**n**	**%**	**n**	**%**	**n**	**%**	**n**	**%**
Colorectal Cancer	1409	10.2	11127	23.1	5678	23.6	3151	19.3
Lung Cancer	2087	15.1	9338	19.4	2624	10.9	1521	9.3
Breast Cancer	754	5.5	5432	11.3	2689	11.2	1660	10.2
Ischaemic Heart Disease	7111	51.6	17268	35.9	10384	43.2	7809	47.8
Cerebro-Vascular Disease	2420	17.6	4963	10.3	2674	11.1	2181	13.4
**Total**	**13,781**	100	**48,128**	100	**24,049**	100	**16,322**	100

## References

[B1] Duckett SJ (2000). The Australian health care system.

[B2] Schoen C, Davis K, DesRoches C, Donelan K, Blendon R (2000). Health insurance markets and income inequity: Findings from an international health policy survey. Health Policy.

[B3] MacLeod MCM, Finlayson AR, Pell JP, Findlay IN (1999). Geographic, demographic, and socioeconomic variations in the investigation and management of coronary heart disease in Scotland. Heart.

[B4] Gregory PM, Malka ES, Kostis JB, Wilson AC, Arora JK, Rhoads GG (2000). Impact of geographic proximity to cardiac revascularization services on service utilization. Med Care.

[B5] Jones AP, Bentham G (1997). Health service accessibility and deaths from asthma in 401 local authority districts in England and Wales, 1988–92. Thorax.

[B6] Sexton P, Sexton TLH (2000). Excess coronary mortality among Australian men and women living outside the capital city statistical divisions. Med J Aust.

[B7] Payne N, Saul C (1997). Variations in use of cardiology services in a health authority: comparison of coronary artery revascularisation rates with prevalence of angina and coronary mortality. BMJ.

[B8] Jong KE, Smith DP, Yu XQ, O'Connell L, Goldstein D, Armstrong BK (2004). Remoteness of residence and survival from cancer in New South Wales. MJA.

[B9] Turrell G, Mathers CD (2000). Socioeconomic status and health in Australia. Med J Aust.

[B10] Clewer A, Perkins D (1998). Economics for Health Care Management.

[B11] Campbell NC, Elliott AM, Sharp L, Ritchie LD, Cassidy J, Little J (2002). Impact of deprivation and rural residence on treatment of colorectal and lung cancer. Br J Cancer.

[B12] Hall S, Holman CDJ, Platell C, Sheiner H, Threlfall T, Semmens J (2005). Colorectal cancer surgical care and survival: do private health insurance, socioeconomic and locational status make a difference?. ANZ J of Surgery.

[B13] Brown IM, Wilson CR, Doughty JC, Weiler-Mithoff EM, George WD (2003). Re: Inequalities in breast cancer reconstructive surgery according to social and locational status in Western Australia (Correspondence). Eur J Surg Oncol.

[B14] Thomson CS, Hole DJ, Twelves CJ, Brewster DH, Black RJ (2001). Prognostic factors in women with breast cancer: distribution by socioeconomic status and effect on differences in survival. J Epidemiol Community Health.

[B15] Holman CDJ, Bass AJ, Rouse IL, Hobbs MST (1999). Western Australia: Development of a health services research linked database. Aust N Z J Public Health.

[B16] Australian Bureau of Statistics (1996). 1996 Census of population and housing: Socio-economic indexes for areas. Canberra: ABS.

[B17] Commonwealth Department of Health and Aged Care (2001). Measuring remoteness: Accessibility/remoteness index of Australia (ARIA). Revised edition. Information and Research Branch Occasional Papers: New Series Number 14.

[B18] Commonwealth Department of Health and Aged Care (2001). Health services in the city and the bush: measures of access and use derived from linked administrative data. Portfolio Strategies Division Occasional Papers: New Series Number 13.

[B19] Charlson ME, Pompei P, Ales KL, MacKenzie CR (1987). A new method of classifying prognostic comorbidity in longitudinal studies: Development and validation. J Chronic Dis.

[B20] Walker A, Thurecht L, Pearse J, Harding A (2003). Hospitalisation rates and costs by socioeconomic status, New South Wales, 1996–97 and 2000–01. Paper presented at the Health Services and Policy Research Conference: 2003 Melbourne.

[B21] Coory M, Scott IA, Baade P (2002). Differential effect of socioeconomic status on rates of invasive coronary procedures across the public and private sectors in Queensland, Australia. J Epidemiol Community Health.

[B22] Hall S, Holman CDJ, Sheiner H, Hendrie D (2004). The influence of socio-economic and locational disadvantage on survival after a diagnosis of lung or breast cancer in Western Australia. J Health Serv Res Policy.

[B23] Campbell NC, Elliott AM, Sharp L, Ritchie LD, Cassidy J, Little J (2001). Rural and urban differences in stage at diagnosis of colorectal and lung cancers. Br J Cancer.

[B24] Segal L (2004). Why it is time to review the role of private health insurance in Australia. Aust Health Rev.

[B25] Stoelwinder JU (2002). The price of choice: Private health insurance in Australia. Aust Health Rev.

[B26] Hopkins S, Kidd MP (1996). The determinants of the demand for private health insurance under Medicare. Appl Econ.

[B27] Palangkaraya A, Yong J (2005). Effects of recent carrot-and-stick policy initiatives on private health insurance coverage in Australia. Econ Rec.

